# Translation and validation of the Korean version of PROMIS® pediatric and parent proxy measures for emotional distress

**DOI:** 10.1186/s41687-019-0120-7

**Published:** 2019-06-20

**Authors:** Heeseung Choi, Chanhee Kim, Heesung Ko, Chang Gi Park

**Affiliations:** 1 College of Nursing and the Research Institute of Nursing Science, Seoul National University, 103 Daehak-ro, Jongno-gu, Seoul, 03080 South Korea; 2 The Research Institute of Nursing Science, Seoul National University, 103 Daehak-ro, Jongno-gu, Seoul, 03080 South Korea; 3 Jesus University, 383, Seowon-ro, Wansan-gu, Jeonju, 54989 South Korea; 4 College of Nursing, University of Illinois at Chicago, Chicago, IL, USA. 845 S Damen Ave, Chicago, IL 60612 USA

## Introduction

Public awareness of mental health among adolescents is growing, as about 10–20% of adolescents suffer from mental health problems globally [1, 2]. Patient-reported outcomes (PROs) are especially important in measuring mental health symptoms such as depression, anxiety, and anger, which are the most common forms of emotional distress experienced by adolescents [3–5]. In addition, parent-proxy reports are also crucial to complement adolescents’ self-report in assessing such emotional distress [6].

The Patient-Reported Outcome Measurement Information System (PROMIS®) was developed by the National Institute of Health (NIH) to address the need for more valid, reliable, and generalizable measures to assess critical PROs [7, 8]. The PROMIS measures efficiently assess health status across populations based on item response theory (IRT). The PROMIS pediatric self-report and parent-proxy item banks have been developed to assess physical, mental, and social health in youth aged 8 to 17 years [9–12]. The PROMIS Pediatric and Parent Proxy scales were adapted and validated in different languages, showing good psychometric properties [13, 14].

Given the PROMIS measures’ advantages in assessing PROs within an innovative framework, we judged it important that these benefits were made available to assess emotional distress in Korean adolescents. Thus, this study aimed to develop a Korean version of the PROMIS Pediatric self-report and parent-proxy measures for emotional distress (i.e., depression, anxiety, and anger) for adolescents and to evaluate their psychometric properties and unidimensionality.

## Methods

### Translation

The translation of the PROMIS Pediatric and Parent Proxy measures for emotional distress was performed after obtaining approval from the institutional review boards at S. University and S. Hospital in Korea. Specifically, we translated the original PROMIS® Pediatric self-report Item Bank Version 1.1 for Depressive Symptoms (13 items), Anxiety (13 items), and Anger (5 items); Parent-Proxy Item-Bank Version 1.1 for Depressive Symptoms (13 items) and Anxiety (13 items); and Parent-Proxy Short-Form Version 1.0 for Anger (5 items) based on standard translation methodology (i.e., Functional Assessment of Chronic Illness Therapy) [15]. The process included forward-translation, reconciliation, back-translation, and expert reviews. The English version of each measure was translated into Korean by two independent bilingual translators. Subsequently, a third bilingual translator reconciled the two previous translations. A fourth independent bilingual translator then performed a back-translation of the reconciled version. Next, three experts (a linguistic expert, a mental health specialist, and an expert experienced in the translation and validation of measures) independently reviewed all the translation steps for each item and selected the most appropriate translation or suggested an alternative one. Finally, the entire translation history for each item was sent to the PROMIS Statistical Center for harmonization and quality assurance to assess their equivalence with the original versions.

#### Cognitive interviews

Cognitive interviews were performed to ensure cross-cultural equivalence between the original and Korean versions and identify possible sources of response errors. For the pediatric measures, a convenience sample of five adolescents aged 13 to 17 years (2 boys; 3 girls) was interviewed. In addition, five mothers of adolescents were interviewed regarding the proxy measures. During the interviews, participants first responded to all the translated items in writing and then, reviewing one item at a time, were asked about difficulties in understanding the item. The meanings of original and translated items were discussed with participants to ensure their equivalence and comprehensibility. After completing all the interviews, the research team analyzed participant comments to evaluate the comprehensibility and equivalence of the translated items. Several translated items that were reported to sound unnatural or vague, and items that used double negatives were revised. Overall, the participants reported no serious difficulties in understanding the translated items.

### Psychometric testing

#### Participants

The psychometric testing included a non-clinical sample from one middle school and two high schools and a clinical sample from one hospital, all in Seoul. First, an information sheet stating the study purpose and procedures was provided to students aged 13 to 17 years and their parents, after getting permission from the school principals. When both students and parents expressed willingness to participate, the participants were each given individual sealed envelopes containing informed consent forms and the Korean version of the PROMIS questionnaires. A total of 160 adolescent-parent dyads completed and returned the consent forms and questionnaires between September and December 2016.

Subsequently, 114 adolescent-parent dyads recruited from the psychiatric outpatient department between December 2017 and March 2018 also completed the consent forms and questionnaires. Eligibility criteria were as follows: (1) aged 12 to 17, (2) able to speak Korean and understand the study purpose and procedures, and (3) no psychotic symptoms or developmental disorders based on the medical records.

#### Measures

Responses to the Korean version of the PROMIS measures were based on recollection of the previous seven days. The measures employed a 5-point Likert scale ranging from 0 (*never*) to 4 (*almost always*) with higher scores reflecting higher levels of emotional distress. The standardized mean score (T-score, with a mean value of 50 and a standard deviation of 10) for each measure was obtained from the online PROMIS Assessment Center.

#### Analyses

Data analyses were performed using STATA version 15 (Stata Corp LP, College Station, Texas, USA) and *R* version 3.5.0. Reliability was examined using Cronbach’s α. Construct validity was measured using correlations between the pediatric and proxy measures for depressive symptoms, anxiety, and anger.

The unidimensionality of the measures was examined through confirmatory factor analysis (CFA), exploratory factor analysis (EFA), omega hierarchical (ω_h_), and explained common variance (ECV). First, one-factor CFA was used, and the comparative fit index (CFI), the Tucker-Lewis Index (TLI), and the root mean square error of approximation (RMSEA) were evaluated to assess model fit. The generally accepted criteria for unidimensionality include CFI > 0.95, TLI > 0.95 [16, 17], and RMSEA < 0.08 [18]. Then, an EFA was performed. Unidimensionality is considered to be supported if a single factor accounts for more than 20% of the variance and the ratio of the first and second eigenvalues is higher than 4 [19]. As an additional approach for assessing unidimensionality, we employed the estimated ω_h_, which is the proportion of total variance attributable to a general factor, using the *Psych* package in *R*. An ω_h_ value of 0.70 or greater indicates that an item set is sufficiently unidimensional for IRT models [20]. Additionally, explained common variance (ECV) was calculated to assess the relative strength of one general factor compared to group factors, and we considered ECV values greater than 0.60 to be acceptable [20].

## Results

Of 274 adolescent-parent dyads who returned consent forms and questionnaires, we excluded 22 dyads with missing data from the analysis. Consequently, data from 252 parent-child dyads were analyzed. In this study, 52.78% of adolescent participants were girls, and 87.30% of parents were women. Mean age was 15.18 years for adolescents and 45.61 for parents (Table 1). Descriptive values of all PROMIS measures are presented in Table 2.

**Table 1:** Participant Characteristics

Characteristics	Non clinical (*n* = 160)	Clinical (*n* = 92)	Total (*N* = 252)
Adolescents			
Gender, n (%)			
Male	74 (46.25)	45 (48.91)	119 (47.22)
Female	86 (53.75)	47 (51.09)	133 (52.78)
Age, mean (SD)	15.14 (1.20)	15.25 (1.65)	15.18 (1.38)
Parents			
Gender, n (%)			
Male	26 (16.25)	6 (6.52)	32 (12.70)
Female	134 (83.75)	86 (93.48)	220 (87.30)
Age, mean (SD)	45.06 (6.53)	46.15 (5.23)	45.61 (5.88)

**Table 2:** Descriptive statistics for PROMIS measures

Measures	Non clinical mean (SD)	Clinical mean (SD)	Total mean (SD)
Pediatric			
Depression (13 items)	49.78 (9.79)	57.65 (10.93)	52.65 (19.88)
Anxiety (13 items)	46.33 (10.52)	54.89 (12.89)	49.45 (12.14)
Anger (5 items)	43.22 (10.71)	49.66 (13.89)	45.57 (12.33)
Parent Proxy			
Proxy Depression (13 items)	48.53 (8.27)	61.81 (8.49)	53.38 (10.51)
Proxy Anxiety (13 items)	45.16 (8.81)	59.27 (10.50)	50.28 (11.63)
Anger (5 items)	41.97 (9.95)	52.03 (11.82)	45.64 (11.70)

Cronbach’s alpha for the pediatric depression, anxiety, and anger measures was 0.93, 0.94, and 0.91, respectively; and for the parent proxy measures, 0.95, 0.96, and 0.90, respectively. Strong relationships were observed between pediatric depression, anxiety, and anger measures (0.70 ≤ r ≤ 0.79; *p* < 0.001) and the respective parent proxy measures (0.59 ≤ r ≤ 0.84; p < 0.001).

The results obtained for unidimensionality are summarized in Table 3. CFA indices outside the recommended ranges were found for pediatric Depression (CFI = 0.92; TLI = 0.91; RMSEA = 0.10), Anxiety (CFI = 0.91; TLI = 0.90; RMSEA = 0.10), and Anger (CFI = 0.96; TLI = 0.91; RMSEA = 0.18). Similarly, indices outside the ranges were observed for parent-proxy Depression (CFI = 0.93; TLI = 0.92; RMSEA = 0.10), anxiety (CFI = 0.89; TLI = 0.86; RMSEA = 0.14), and anger (CFI = 0.97; TLI = 0.95; RMSEA = 0.13). Although the CFA indices were slightly beyond the recommended ranges, the EFA results supported unidimensionality. The first factor of pediatric Depression, Anxiety, and Anger explained 94.52%, 94.23%, and 100% of the variance, respectively. Additionally, the ratios of the first and second eigenvalues for pediatric Depression, Anxiety, and Anger were greater than 4. In the case of the parent-proxy measures, the first factor of proxy-report Depression, Anxiety, and Anger explained 95.38%, 92.88%, and 100% of the variance, respectively, and the ratios of the first and second eigenvalues were greater than 4 as well. In addition, values of estimated omega hierarchical (ω_h_) for pediatric Depression, Anxiety, and Anger were 0.83, 0.81, and 0.84, respectively, and those for parent-proxy Depression, Anxiety, and Anger were 0.88, 0.87, and 0.87, respectively. All the omega hierarchical (ω_h_) values for pediatric and parent proxy measures were higher than 0.7 and thus supported unidimensionality. Furthermore, the ECV values for the pediatric and parent-proxy measures were greater than 0.6, thus indicating unidimensionality. Based on these results, the Korean PROMIS Pediatric self-report and parent-proxy measures sufficiently exhibited unidimensionality.

**Table 3:** Model fit indices for Depression, Anxiety, and Anger

Measures	CFI	TLI	RMSEA	Omega Hierarchical	ECV
Pediatric					
Depression (13 items)	0.92	0.91	0.10	0.83	0.80
Anxiety (13 items)	0.91	0.90	0.10	0.81	0.74
Anger (5 items)	0.96	0.91	0.18	0.84	0.83
Parent Proxy					
Proxy Depression (13 items)	0.93	0.92	0.10	0.88	0.85
Proxy Anxiety (13 items)	0.89	0.86	0.14	0.87	0.81
Anger (5 items)	0.97	0.95	0.13	0.87	0.85

Note. ECV Explained Common Variance of the general factor

## Discussion

This study was conducted to translate the PROMIS Pediatric and Parent-Proxy measures for emotional distress into Korean and evaluate their psychometric properties among Korean adolescents. The Korean version of the PROMIS Pediatric self-report and parent-proxy measures for emotional distress for adolescents were developed based on the standard PROMIS methodology and demonstrated satisfactory psychometric properties and unidimensionality, similar to those reported in previous studies [21–24].

The PROMIS measures for emotional distress have been found to be easy to understand and complete with minimal burden and can be used in both research and clinical settings. In addition, the use of self-report and parent-proxy measures may enable researchers and clinicians to gain a deeper understanding of adolescents’ emotional distress and assess the association between parents’ and adolescents’ perceptions of it.

As for study limitations, our convenience sample consisted of adolescents recruited from schools and a hospital in Seoul, potentially compromising the generalizability of our findings. Thus, additional studies with larger samples, more varied populations, and pre-adolescent participants are called for. Moreover, cross-cultural validation requires the use of differential item functioning for Korean and English measures. Finally, this cross-sectional study could not assess the predictive validity of the emotional distress measures.

In conclusion, the Korean version of the PROMIS Pediatric self-report and parent proxy measures for emotional distress are reliable and valid for Korean adolescents and their parents. Given the increasing prevalence and seriousness of adolescents’ mental health problems, the Korean version of PROMIS measures could advance early detection and intervention for adolescents’ mental health problems.

## Abbreviations

CFA: Confirmatory factor analysis
ECV: Explained common variance
EFA: Exploratory factor analysis
IRT: Item response theory
PROMIS®: Patient-Reported Outcome Measurement Information System
PROs: Patient-reported outcomes
RMSEA: Root mean square error of approximation
ω_h_: Omega hierarchical
